# Rapid Deployment of National Guard Alternative Healthcare Facility With Isolation Unit Capabilities in Response to COVID-19

**DOI:** 10.1093/milmed/usaa546

**Published:** 2020-12-09

**Authors:** Jacob Torrey, Jeremy Orr, Jonathon Florance

**Affiliations:** Medical Service Corps Vermont ARNG, Geisel School of Medicine at Dartmouth College, Hanover, NH 03755, USA; SP Corps Vermont ARNG, Envision Healthcare LLC, Portsmouth Regional Hospital, Portsmouth, NH 03801, USA; Medical Corps Vermont ARNG, Department of Orthopaedic Surgery, Duke University Medical Center, Durham, NC 27710, USA

## Abstract

At the direction of Governor Phil Scott, the Vermont National Guard rapidly erected a 400-bed alternative healthcare facility field hospital to increase the state’s medical capacity early in the COVID-19 pandemic when information was limited and cases were rapidly rising across the country. This case study reviews the preparation and management of the alternative healthcare facility’s first COVID-19-positive patient assigned to the 50-bed COVID-19 isolation ward. Despite austere conditions with rudimentary improvements to a nonstandard facility, the ad hoc team composed entirely of members of the Vermont National Guard successfully oversaw patient care from admission to discharge while maintaining a zero-percent transmission rate to staff. While the local civilian medical infrastructure was never overwhelmed and patient census at the facility remained low, this case study highlights the capability of the National Guard enterprise as a community response to pandemic crises.

## INTRODUCTION

In March 2020, the Vermont Governor’s Office initiated coordination with the Vermont National Guard (VTNG) in anticipation of a worst-case scenario in the escalating COVID-19 pandemic. Modeling suggested that the Vermont COVID-19-positive population requiring hospitalization could drastically exceed the number of available hospital beds in the state.^1^ The governor ordered the VTNG to assist by erecting and staffing a 400-bed alternative healthcare facility (AHF) field hospital at the Champlain Valley Exposition Center.^2^ A 50-bed isolation ward of the AHF was designated to manage COVID-19-positive patients. To fulfill all requirements of this mission—ranging from daily clinical operations to command and control—the VTNG created the Joint Medical Task Force (JMTF), an ad hoc team formed entirely from the Vermont Army National Guard and Vermont Air National Guard.

**FIGURE 1.**
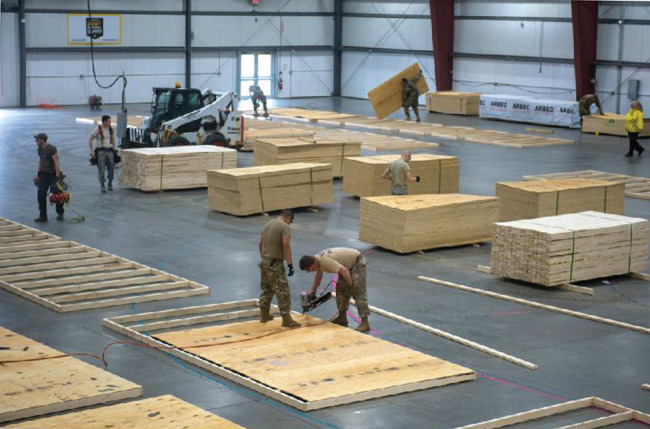
Alternate healthcare facility construction.

**FIGURE 2.**
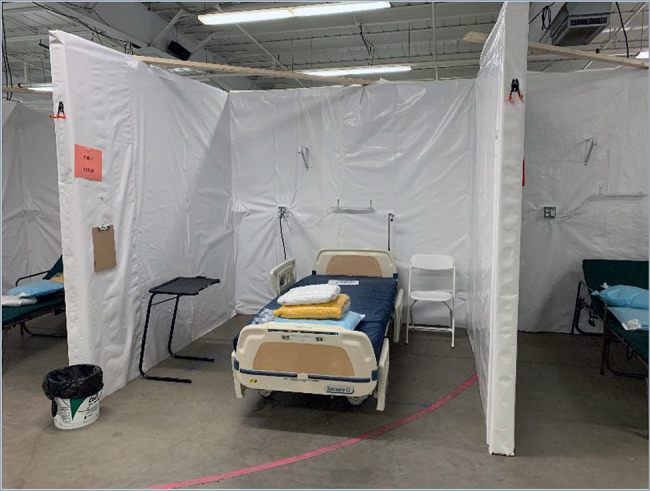
Alternate healthcare facility isolation ward patient bed.

**FIGURE 3.**
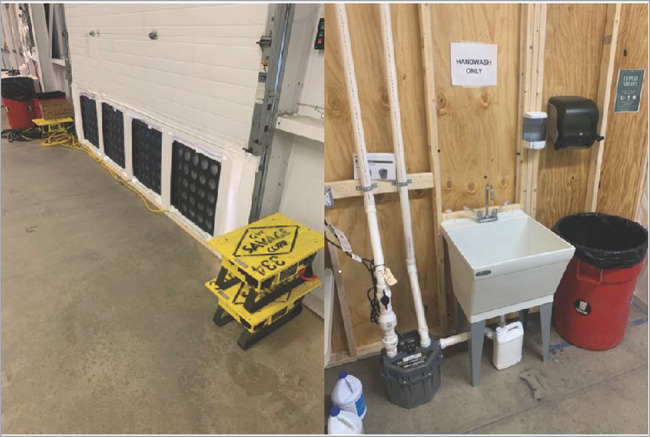
Alternate healthcare facility modifications included industrial fans for negative pressure airflow and additional hand-washing stations.

The AHF was erected within the existing Champlain Valley Exposition Center outside of Burlington, Vermont, in Chittenden County. This location was geographically ideal, being the most populous area of the state and with the greatest number of COVID-19 cases. The nonstandard facility consisted of several connected metal-framed buildings totaling 7,500 square meters (81,000 square feet). There were central bathrooms with indoor plumbing, but the majority of the indoor area was open with concrete slab flooring. Fig. 1 pictures early construction. In under 2 weeks, Vermont state employees and National Guard members transformed the space with wood framing, power, plumbing, and requisite supplies to provide a combination of semi-private and open-bay hospital beds divided across eight functional wards. The isolation ward was established in a separate building, connected by a large hallway to the remainder of the facility. Guarded checkpoints were established to prevent unauthorized movement from the larger facility. Additional hand-washing stations—straddled on either side by partitioned doorways—created a staging area known as the “Warm Zone” before entering the isolation ward. Private patient toilets and showers were constructed in the isolation area to minimize contact and cross contamination of the virus. A negative pressure environment was created in the isolation ward with the use of industrial fans with appropriate filtration and vented to the outside. Figs. 2 and 3 picture the facilities with selected improvements.

The AHF was equipped to manage low-acuity patients, providing all services that a skilled nursing center would provide. Utilizing a full range of military occupational specialties within the VTNG, the AHF maintained 24-hour medical provider coverage, an in-facility rapid response team, registered nurses managing a team of medics on each ward, an on-site pharmacy, portable radiograph capability, patient laboratory draws, and the ability to provide daily physical therapy. There were physical and technological limitations that rendered the Essex AHF dependent on the University of Vermont Medical Center (UVMMC) for advanced diagnostic capabilities and higher level of care if required. Joint Medical Task Force personnel serving as liaisons, patient affairs, and case managers were intimately integrated with the UVMMC to enable smooth transition between facilities. The administrative handoff was important since the AHF primarily used paper medical records.

The entire facility and grounds had a mandatory mask requirement, and social distancing was enforced regardless of personal protective equipment (PPE) use. Staff occupied the entrance checkpoint 24 hours a day and were responsible for maintaining a movement ledger of personnel. Additionally, all personnel submitted to daily temperature checks and screening questions. Though there was a no visitor policy, patients were able to interact with family members via cell phone or devices connected to a local Wi-Fi.

Vermont Governor Phil Scott and the University of Vermont Division of Infectious Disease inspected the AHF before opening. On April 5, the AHF was determined to be operational, meeting all essential regulations of the Vermont Agency of Human Services. On April 10, the AHF received its first patient, PM.

## THE VTNG ALTERNATE HEALTHCARE FACILITY

**FIGURE 4.**
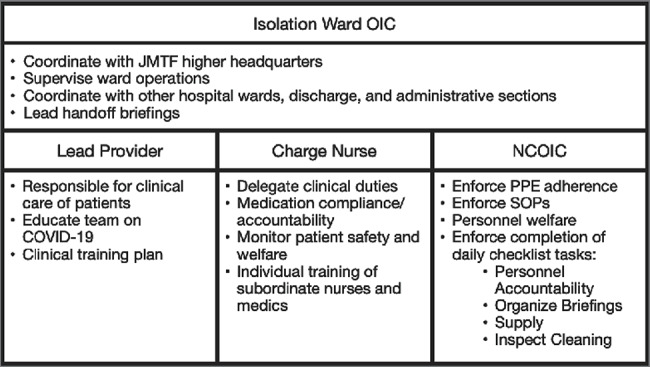
Alternate healthcare facility ward leadership roles.

In managing patients with acute COVID-19 infections in a nonstandard environment, the AHF staff leveraged military experience to quickly organize while minimizing the risk of transmission. Given the need to minimize large groups across the organization, these efforts relied on a clear chain of command and initiative of lower-level leaders. The isolation ward Officer in Charge coordinated with JMTF leadership and led the ward’s daily handoff briefings. The Officer in Charge collaborated with the clinical team to develop appropriate risk mitigation measures for the ward that included ward layout design and movement protocols for patients, staff, and materials. The lead provider administered updates on COVID-19 and spearheaded education. The charge nurse outlined major clinical duties for the shift. The Non-Commissioned Officer in Charge enforced PPE adherence, isolation ward standard operating procedures (SOPs), and the completion of daily task checklists. Fig. 4 summarizes the roles and responsibilities of ward leadership.

Utilizing risk analysis techniques common to military organizations, isolation ward SOPs established a baseline that kept the staff operating safely. All personnel entered the ward at the “Cold Zone,” where personnel left personal belongings. No unauthorized personnel were allowed beyond the “Cold Zone,” which served as the drop point for supplies. In the “Warm Zone,” personnel would exchange surgical face masks for N95 face masks and then wash hands at the mandatory station. N95 use was mandatory at all times in the “Hot Zone.” Inside the “Hot Zone,” patients were in semi-private rooms of three walls, which opened to a central corridor. To enter the corridor, staff members would don a Tyvek suit with hood, two pairs of gloves, face shield, and boot covers.

In addition to stratifying the ward physically into risk zones, leadership minimized staff turnover to decrease opportunities for lapses in safety. Medics and nurses would frequently be in Tyvek suits for hours to minimize the number of transitions in and out of PPE. Staff members worked longer and more frequent shifts than typical employment—frequently every day for 12 hours—minimizing the number of exposed staff members. Additionally, a smaller team meant a more cohesive understanding of ward culture. As the patient census on the ward grew, new staff members would be trained and shadowed to ensure adherence to internal SOPs.

Lastly, the ties to community inherent to a National Guard response enabled extensive reach to local assets outside of the AHF. Case management communicated daily to outside facilities, an effort that became paramount as skilled nursing facilities refused admission of COVID-19-positive patients and later increased screening requirements. Additionally, medical provider and nursing connections to nearby UVMMC enabled cross communication that would have been impossible with teams from outside the network. These connections proved valuable during patient transfers from the UVMMC and for the identification of resources.

## CASE PRESENTATION

PM was a 39-year-old male who was transferred to the AHF from a community group home with a positive COVID-19 test result after known exposure to an infected individual. PM had no symptoms upon presentation. He denied cough, shortness of breath, sore throat, chest pain, myalgia, fatigue, headache, nausea, vomiting, diarrhea, anosmia, or ageusia. The patient agreed to care at the AHF as his best option for quarantine given social constraints.

His past medical history was notable for depression, attention deficit hyperactivity disorder, generalized anxiety disorder, PTSD, and substance use disorder. His past surgical history was noncontributory but included multiple left knee surgeries from traumatic anterior cruciate ligament rupture. The patient arrived to the AHF with a 14-day supply of mirtazapine 30 mg nightly, hydroxyzine 50 mg three times a day, and buprenorphine/naloxone 14 mg daily.

Despite endorsing no symptoms and recent confirmation of being afebrile, PM arrived to AHF febrile with a temperature of 38.6°C (101.5°F). Otherwise, vital signs were normal. The patient was hemodynamically stable and appeared to be well nourished and in no acute distress. His lungs were clear bilaterally to auscultation, and his abdomen was nontender.

With compliance and modification to the initially established SOPs of the VTNG AHF, patient PM was continued on his home medications and prescribed 650 mg acetaminophen as needed for fever. The frequency of vital signs was decreased to daily as the patient was deemed medically stable. The provider examined the patient daily but determined minimal incremental change in his course. PM remained intermittently febrile and infrequently endorsed sweating and lower extremity myalgia, but was otherwise asymptomatic. The patient responded well to acetaminophen but chose to abstain from consistent antipyretics to determine eligibility for housing. Upon cessation of his fever for 72 hours without the use of antipyretic medication, the patient was discharged.

The AHF ultimately accepted 20 patients, 7 of them tested COVID-19 positive and 13 COVID-19 negative. The largest census on the isolation ward at any one time was five patients. The majority of COVID-19 care consisted of supplemental oxygen, antipyretic medication, and increasing mobility through physical therapy. Notably, many patients were physically dependent on others, so staff were intimately involved with activities of daily living. No soldiers or airmen assigned to either the isolation or nonisolation ward developed signs and symptoms for COVID-19 or tested positive for COVID-19 despite daily, intimate contact. No family members or cohabitants of these soldiers and airmen developed symptoms or tested positive for COVID-19. There was no cross contamination of COVID-19 to the other patients in the isolation and nonisolation wards.

In the spring and summer of 2020, the number of patients in Vermont never exceeded civilian hospital capacity and the census at the AHF remained relatively low. Given improving projections of community infection, the AHF stopped accepting patients and subsequently closed. Portions of the field hospital were deconstructed and stored, whereas another portion remained in place in case of a possible resurgence of COVID-19. While the government’s decision to employ forces in this case proved to be an action of preparation that was ultimately not needed on the anticipated scale, the AHF successfully provided quality care to patients and served as a proof of concept for expanding hospital capacity.

In leveraging the National Guard for expanding hospital capacity by decongesting civilian facilities of low-acuity but labor-intensive patients, the following sampling of lessons learned would be valuable for future missions:

- Rehearse to overcome ad hoc teams: Each JMTF ward was an ad hoc team, comprised of soldiers and airmen from a variety of elements. Doctrinally, such teams can lead to poor performance, but the JMTF was successful by incorporating time to develop internal SOPs before beginning operations. Rehearsals were conducted across the facility to develop group norms.
- Balance increased span of control with additional staff resources: The JMTF was comprised of eight medical wards in addition to elements including triage, discharge, and patient administration. This task organization was dictated by the mission; however, it placed incredible strain on the staff who coordinated the many subordinate elements. In establishing a JMTF for a large field hospital, allocating sufficient staff assets will enable smoother operations with special attention paid to logistics.
- Risk mitigate delivery of medication: Administering medication was the most dangerous function of the AHF. Civilian medical facilities institute considerable electronic and engineered safety mechanisms to minimize human error while distributing medication, but many of these systems are not feasible in a field hospital. The AHF relied on rehearsed SOPs, nursing continuity to minimize handoff error, and secondary observers during medicine dispensation to ensure accountability.
- Robust physical therapy capability: The majority of the patients accepted to the AHF required substantial assistance with activities of daily living, precisely because these were the patients that congested the civilian medical system. Daily or twice-daily physical therapy was the norm at the AHF through the use of a physical therapist who trained and oversaw a staff of orderlies who had minimal previous medical training.

## LITERATURE REVIEW

Worldwide, the medical community has found numerous ways to expand healthcare capacity in response to crises. Multiple countries have employed their military forces to fight COVID-19, finding that military medicine is uniquely suited to respond to such a rapidly evolving and large crisis. Temporary hospitals supporting treatment of epidemic diseases have precedent, including notable examples in the United States, France, and China in support of the current fight against COVID-19, as well as previous examples during Ebola virus disease (EVD) outbreaks.

COVID-19 initially emerged in Wuhan, China, and exponential growth of cases there quickly overwhelmed that city’s existing medical infrastructure.^3^ One response taken by the Chinese government was to create temporary hospitals in existing nonmedical buildings, known as fangcang hospitals. These facilities were intended to isolate and provide basic medical care to asymptomatic and mild cases of COVID-19 and provide services such as monitoring and rapid referral while meeting three criteria of rapid deployment, massive scale, and low cost.^4^

The experience in China demonstrates the speed and cost savings involved in repurposing existing nonmedical facilities. The Chinese government also built new facilities to address the surge of cases and found those facilities to be 10 times more expensive than the fangcang model.^5^ Although fangcang hospitals bear many similarities to the VTNG AHF, a key difference is that the fangcang hospitals were built with a different purpose, intended to isolate both mild and entirely asymptomatic cases of COVID-19 in order to prevent community and family spread. In the United States, CDC guidance has consistently advised that individuals that have asymptomatic to moderate disease isolate at home, and only moderate to severe cases should seek hospital care.^6^ The result is that U.S. facilities, such as the AHF, are not scaled as large or replicated as often as in China.

The Chinese efforts were largely successful, but not without difficulties. Organized at a national level, the fangcang hospitals brought in staff members from across China, resulting in new and untested organizational structures and relationships.^7^ A lack of managerial skills and experience in the teams leading the fangcang hospitals resulted in confusion among staff members regarding roles, responsibilities, and SOPs.^8^ Bringing together medical staff and ancillary staff with no preexisting relationship added additional challenges in training and enforcing infection control protocol, an aspect vital to protecting staff and keeping the hospitals functioning and open.^5^

Recognizing the unprecedented need to rapidly expand healthcare capacity and the resources and training of military forces, many nations have employed their military medical capabilities to address COVID-19. Military forces provide a formidable capability in crisis response, especially when the military is doctrinally trained to employ mission command. Mission command is a military concept that gives subordinate units and individuals the freedom of action to take initiative within the commander’s intent. Mission command is a cornerstone of successful military operations and is key to why military medicine reacts to unprecedented crises quickly and effectively.^9^

A notable recent historical example of military medicine responding to a highly infectious disease is the UK military’s response to the Sierra Leone EVD outbreak in 2014-2015. This response demonstrates how using rigorous military planning processes, similar to the U.S. Army’s Military Decision Making Process, can lead to success on high-risk medical missions.^10^ By investing time and resources into planning and training, the UK military medical unit was able to identify and mitigate the high risk inherent in treating EVD patients in an austere environment. They succeeded by identifying hazards, creating risk mitigation factors for those hazards, and then training all personnel on using those risk mitigation factors. This was followed by enforcing and supervising the risk mitigation procedures, while retaining the flexibility to adjust as the situation evolved.^11^

Recent literature demonstrates the use of regular military units in support of the COVID-19 response. In addition to deploying existing field hospitals to New York City and Seattle, the U.S. Army established a large AHF at the Javits Center in New York City, which treated 1,095 patients, with a maximum daily census of 453 patients. During the course of operations, only 11 personnel were infected with COVID-19. Although the leadership initially encountered friction in organizing this broad effort, they prevailed by empowering experienced and effective leaders and by implementing a robust liaison program with local hospitals.^12^

France responded to COVID-19 by mobilizing all departments of the French Military Medical Service. This was a multifaceted effort that included advising civilian systems, supporting logistics, and increasing hospital capacity in key areas. The French Military Medical Service successfully deployed a tent field hospital within France, adding 30 staffed ICU beds. They successfully mitigated risk and prevented the spread of infection within the field hospital by implementing three zones that each required personnel to don a different level of PPE.^13^

## CONCLUSION

This case report demonstrates the efficacy of the National Guard enterprise in managing temporary hospitals treating low-acuity COVID-19-positive patients. Although the number of hospitalized patients was ultimately limited, all episodes ended with successful patient discharge, no transmission of COVID-19 to the staff, and universally excellent ratings reported from patient surveys. The results indicate that the National Guard is well suited for the task of organizing this type of response precisely because of its intersection of military culture with community connections.

With the ongoing Global War on Terror approaching 20 years in length, the National Guard has amassed significant experience in operating as teams to accomplish missions in chaotic environments. These experiences have forged an organization with institutional management and risk mitigation skills that leverages military values to succeed in any environment. These bedrock capabilities enabled the VTNG to manage an ad hoc team while succeeding in a completely different kind of mission.

The community connection of the National Guard makes the organization inherently different from the active duty military. The majority of the organization is composed of service members who wear a uniform part time while balancing a career in the local civilian community. Those community ties add incredible value when operating in the highly interconnected world of medicine, where the team must understand the environment and work with local agencies to best care for patients.
